# Psychometric characteristics of the “General Oral Health Assessment Index (GOHAI) » in a French representative sample of patients with schizophrenia

**DOI:** 10.1186/s12903-017-0368-3

**Published:** 2017-04-11

**Authors:** Frederic Denis, Mohamad Hamad, Benoit Trojak, Stéphanie Tubert-Jeannin, Corinne Rat, Jean-François Pelletier, Nathalie Rude

**Affiliations:** 1 La Chartreuse Psychiatric Centre, 1, boulevard Chanoine Kir, BP 23314, 21033 Dijon, Cedex France; 2 EA 481 Integrative Neurosciences and Clinical, University Hospital of Besançon, F-25000 Besançon, France; 3 Department of Dentistry, University Hospital of Dijon, 21079 Dijon, France; 4 Department of Psychiatry and Addiction, University Hospital of Dijon, 21079 Dijon, France; 5 Université Clermont Auvergne, EA4847 CROC Centre for Clinical Research in Dentistry, BP10448, 63000 Clermont-Ferrand, France; 6 Department of Psychiatry, Montreal University, Yale Program for Recovery & Community Health, Montreal, Canada

**Keywords:** Oral health, Quality of Life, Psychometric properties, Schizophrenia, GOHAI

## Abstract

**Background:**

The “General Oral Health Assessment Index” (GOHAI) was widely used in clinical or epidemiological studies worldwide, as it was available for use in different languages. Therefore, the aim of this study was to evaluate the psychometric characteristics of the GOHAI in a representative sample of patients with schizophrenia.

**Methods:**

A total of 90 schizophrenic patients (in-patients and out-patients) were recruited from the participants of the “buccodor study” (NCT02167724) between March and September 2015. They were selected using a random stratified sampling method according to their age, sex, or residential area (urban/rural area). GOHAI validity (construct, predictive, concurrent and known group validity) and internal consistency (reliability) were tested. Test-retest reliability was evaluated in 32 subjects.

**Results:**

The mean age was 47.34 (SD = 12.17). Internal consistency indicated excellent agreement, with a Cronbach’s $\alpha$ value of 0.82 and average inter-item correlation of 0.65. Intraclass correlation coefficients for test-retest reliability with 95% confidence intervals were not significantly different (*p* > 0.05). Construct validity was supported by three factor that accounted for 60.94% of the variance observed. Predictive validity was corroborated as statistically significant differences were observed between a high GOHAI score, which was associated with self-perceived satisfaction with oral health, lower age and high frequency of toothbrushing. Concurrent validity was corroborated as statistically significant relationships were observed between the GOHAI scores and most objective measures of dental status. For known group validity, they was no significant difference of the mean GOHAI score between out or in-patients (*p* > 0.05).

**Conclusion:**

Acceptable psychometric characteristics of the GOHAI could help caregivers to develop ways to improve the Oral Health related Quality Of Life of schizophrenic patients.

**Trial registration:**

Clinical Trials Gov NCT02167724. Date registered 17 June, 2014.

## Background

Schizophrenia is a severe mental disorder that affects 1% of the world population [1, 2] and 600,000 people in France. Schizophrenic patients have excess mortality (their life expectancy is reduced by 20%) and excess morbidity [3]. Among somatic comorbidities in schizophrenia patients, poor oral health has been reported by many authors and contributes to the overall poor health of these patients [4, 5]. Generally, schizophrenia leads to disturbances in the progression of thought, errors in contextual analysis and errors of logic. Often patients with schizophrenia do not recognise their health needs and delay seeking advice or treatment [6].

In dentistry, objective measures of dental diseases do not accurately reflect the patients’ perception of their oral health. Oral Health related Quality Of Life (OHrQOL) questionnaires are interesting tools that evaluate oral health from the patient’s perspective. They take into account the multidimensional aspects of health and consider the functional and psychosocial impacts of dental diseases [7].

In the general population, these OHrQOL scales are used to assess a patient’s condition or a change in oral status during the course of care, and integrate the perceptions and expectations of the patient. A variety of OHrQOl instruments have been developed. For example, the General Oral Health Assessment Index Questionnaire (GOHAI) developed by Atchison and Dolan [8] has been widely used to assess oral health in clinical or epidemiological studies. Validated initially in the USA, this questionnaire is available in French [9]. The GOHAI assesses self-perceived oral health through 12 questions that explore the pain, discomfort, dysfunctions and the psychosocial impacts of dental diseases [10]. It can be self-administered and is quick and easy to use.

The Oral Health Impact Profile-14 (OHIP-14) is also widely used [11]. However, the GOHAI identified more oral functional and psychosocial impacts than did the OHIP-14 [12]. With the OHIP-14 it is more difficult to detect within-subject change [13]. Finally, OHIP-14 is not available in French.

International studies have reported that the oral health status of mentally ill patients was poor compared with that in the normal population [14, 15]. They pay less attention to the presence of conditions such as dental caries and periodontal disease [16]. If left untreated, these conditions can lead to partial or total tooth loss (edentulism), thus compromising nutrition and general physical health. These patients diagnosed with serious mental illness also tend to report perceptions of poor oral health and express important care needs [17, 18]. The Quality-of-Life Scale (QLS) [19] and the Schizophrenia quality of life Scale (S- QOL) [20] have been validated for use in schizophrenic patients, but these tools do not measure OHrQOL. Yet more specific assessments of oral health by caregivers and better management of dental care needs are essential. The implementation of adequate medical or dental care is delayed to the detriment of patients’ health. It is therefore important for caregivers to have access to validated tools to assess the oral health of these patients in order to evaluate accurately their specific needs and to offer appropriate support.

As far as we know, no study has investigated the validity of existing OHrQOL questionnaires in schizophrenic patients.

The aim of this study was to evaluate the psychometric characteristics of the GOHAI scale in a representative sample of patients with schizophrenia.

## Methods

### The GOHAI scale

The GOHAI is a self-assessment oral health questionnaire, which was initially validated for use in elderly North Americans [8]. A French version has been validated in France in a sample of middle-aged adults [9]. The questionnaire consists of 12 questions. Nine negative questions and three positive questions were asked to discourage respondent acquiescence. The 12 questions assess physical functions (eating, talking and swallowing) for items X1, *X*2, X3 and X4 and psychosocial aspects (self-esteem, social withdrawal and worries about oral health) for items X6, X7, X9, X10 and X11. Items X5, X8 and X12 assess symptoms (use of drugs to relieve pain, discomfort) related to the presence of dental diseases.

There are five response categories for each question and a score has been assigned for each response category (l = always, 2 = often, 3 = sometimes, 4 = seldom, and 5 = never). Scores from the positively worded questions were reversed during data processing so that the directions of all responses were the same. The GOHAI score was computed by adding up the scores of the responses to the 12 questions. The GOHAI score is the sum of the answers to the 12 questions so that a high score (Maximum = 60) means satisfactory oral health. The GOHAI scores were ranked in 5 categories ([1-20],[21-30],[31-40],[41-50],[51-60]). There is no recommended cut-off value for the GOHAI to discriminate between good and poor oral health.

### Study population

The sample size recommendations range from 1.2 to 20 subjects per item for exploratory factor analysis (EFA) [21–24] while for Tabachnick and Fidell [25] 300 subjects are needed. There is a lack of consensus regarding how to compute the sample size to assess patient-reported outcome measures [22]. Given the low prevalence of schizophrenia and to ensure the feasibility of the study, we included 90 subjects (7.5 participants per item).

For the present study, 90 patients were recruited from the BUCCODOR survey (NCT02167724). BUCCODOR is a Multicentre cross-sectional study, which evaluated the oral health of patients with schizophrenia in the department of “Côte d’Or” in France (530 000 inhabitants) [26].

The patients were selected and examined between March and September 2015.

They were selected using a stratified randomization procedure, according to their age, sex or residential area (urban/rural area). A list of out- or in-patients schizophrenic persons was drawing up between from the administrative database of the three psychiatric hospitals of the department. They have been selected according to the population characteristics in Côte d’Or. In this French department 30% live in an urban area, 10% in a semi-urban area and 60% live in a rural area according to the National Institute of Statistics and Economic Studies (INSEE) data [26]. A person who refused to participate in this study was replaced by a person with the same characteristics.

In 1960, France opted for mental health care based on the definition of local catchment areas. The country was divided into sectors of approximately 70000 inhabitants [27]. For example, in Côte d’Or there are 7 sectors. The Chartreuse hospital manages 5 sectors (urban/rural area), while the CHU of Dijon (urban area) and the Robert Morlevat hospital (rural area) manage one sector each that includes all adult psychiatric patients. Each sector is run by a multidisciplinary team intended to provide preventive as well as curative and rehabilitative care for all those within the catchment area who need it. Care is provided as close to home as possible. Patients can live in the community and have ongoing contact with the psychiatric outpatient unit or can be hospitalized depending on the severity their mental disorders [27].

Patients who were recruited and accepted to participate were over 18 years of age with a diagnosis of schizophrenia (according to the International Classification of Diseases 10th Revision: ICD-10) [28], and were psychically stable according to a psychiatric evaluation. Exclusion criteria were persons not covered by national health insurance, pregnant or breast-feeding women, persons not stabilized from a psychiatric viewpoint, persons experiencing an acute psychiatric episode, persons who could not understand or had a poor understanding of French.

### Data collection

One investigator interviewed and clinically examined all the participants at the three study sites.

Participants were asked to answer the GOHAI questionnaire by themselves but also to respond to other questions relating to their socio-economic status (SES) (age, sex, level of education, residential area), health status (duration of mental illness,), dental attendance and oral behaviour (smoking habits, tooth brushing frequency). Finally, patients were questioned about their perceived satisfaction with their oral health.

The same day, each patient benefited from a clinical oral examination using portable dental equipment. Pre-packaged and disposable instruments were used. Dental caries was assessed at the dentinal (D3) level, and the Decayed, Missing, or Filled Teeth (DMFT) index, based on 32 teeth, was calculated using WHO (World Health Organization) criteria [29]. The DMFT index is the standardized index to evaluate dental caries with scores ranging from 0 to 32. High scores indicate worse dental health. The mean DMFT in France, in psychiatric inpatients was 15.8 (SD = 8.8) [30]. Dental plaque and calculus were evaluated on six tooth surfaces per participant using the Simplified Oral Hygiene Index (OHI-S) [31]. The mean OHI-S scores were classified into four levels: Excellent (0); Good (0.1-1.2); Fair (1.3-3.0); Poor (3.1-6.0).

### Ethics

This study has been approved by the ethics Committee for the Protection of Persons (CPP) number I of Eastern France (registration number: 2014-A00358-39).

Each patient invited to participate in the study was informed by mail about the objectives of the study. Informed consent was obtained from all the participants (and from their legal guardians for persons under guardianship).

### Data analysis

#### Reliability

The internal consistency of the GOHAI scale and the test-retest reliability were assessed. Internal consistency was assessed by Cronbach’s α coefficient and inter-item correlation [32]. All 90 subjects were included for the internal consistency test. Cronbach's α value > 0.75 indicates excellent reliability whereas values between 0.40 and 0.75 show fair to good reliability, and values of <0.40 indicate poor reliability [32].

Test-retest reliability was assessed by repeating the GOHAI questionnaire in a subset of 32 participants. There is no sample size or power calculation for test-retest, but a general rule of thumb is that 25 to 50 patients make an adequate sample for validation in the data analysis [22].

For the second administration, done three weeks after the first, patients completed the questionnaire on paper and the responses to the items of the GOHAI scale were collected during a telephone call.

Pearson’s Intra class correlation coefficient (ICC) was computed according to Shourt and Fleiss [33]. Values of ICC > 0.80 indicate good agreement, 0.41-0.60 moderate agreement and values <0.40 indicated poor agreement. A Wilcoxon paired test was used to test for significant differences between the two sets of results [34].

#### Validity

Predictive validity was evaluated by examining the association between the GOHAI score and the global question on oral health satisfaction, socio-economic status (SES) (age, sex, level of education, residential area), data relating to oral health (tooth brushing frequency, last dental visit) duration of mental illness and smoking habit. It was hypothesized that SES, behavioural variables and perception in OHrQOL were predictive of GOHAI scores [22, 35].

Concurrent validity was evaluated by comparing the GOHAI scores with the objectively assessed dental status. It was hypothesized that there would be a correlation between the GOHAI scores and the number of decayed, missing and filled teeth (DMFT) or the OHI-S index. It was hypothesized that participants reporting functional problems, psychosocial impacts, pain or discomfort and thus having a low GOHAI score would be more likely to report dissatisfaction with their oral health [22, 35].

Known-groups validity was evaluated by comparing the GOHAI scores between in- and out-patients. It was hypothesized that in-patients would be most affected by the disease and would have a poorer perception in OHrQOl, than out-patients, and that GOHAI would be sensitive to differences between the two groups of patients [22, 35].

As the data was skewed, non-parametric statistics tests were used. The Mann–Whitney *U* test was used to compare the score across the carious, missing and filled teeth, age, sex, level of education, duration of mental illness, dental attendance, oral behaviour variables and the mean GOHAI score. While the Kruskal-Wallis H test was used to evaluate statistical differences between the DMFT, OHI-S and the differential residential area (out- or in-patients). The Jackknife method was used to calculate the confidence intervals.

For construct validity, EFA was used to assess the factor structure of the GOHAI. Due to the small number of participants, we did not perform a confirmatory factor analysis (CFA). Indeed, for CFA, recommendations range from 150 to 1000 subjects [36, 37]. The factor extraction criterion was eigen value more than 1. An item is considered to have loaded onto a factor when the factor loading exceeded 0.30. Prior to the extraction of the factors, The Bartlett’s test sphericity, scree plot, and the Kaiser-Mayer-Olkin (KMO) test were used. The KMO index ranges from 0 to 1, with 0.50 considered suitable for analysis. The Bartlett’s Test of Sphericity should be significant (*p* < 0.05) for factor analysis to be suitable [38, 39].

R Commander® and SAS® Software were used for the statistical analyses.

## Results

### Population characteristics

A total of 264 patients were contacted and 90 of these were included in the study. The average age was 47.34 years (SD = 12.17) (range 21–75). The majority were male (64.4%). The sample’s sex ratio of our study was reflective of the population’s sex ratio. A high proportion had completed high school (77.8%) and were single (74.3%). Most patients (91%) lived at their own home (67.7%) or with their family (23.3%) (out-patients). Only 9% lived in a medico-social institution (in-patients). Our sample is in accordance with the percentage of schizophrenic patients hospitalized since long period in France. The mean DMFT was 16.59 (SD = 8.11) and mean OHI-S was 2.40 (SD = 1.32). Half of the patients (52.2%) had suffered from schizophrenia for more than 18 years and were smokers (53.3%). The majority of patients (64.4%) declared that they brushed their teeth and had not seen a dentist for more than 6 months (60%).

### Acceptability and responses to the GOHAI

The proportion of missing responses varied from 1.1% for item *X*2 to 2.2% for items X3 and X4. Missing data were corrected for by simple imputation of average values [40]. The distribution of the GOHAI scores per item is presented Table 1.

**Table 1:** GOHAI items and frequency distribution of the responses (*n* = 90)

In the past three months	Never	Seldom	Sometimes	Often	Always
How often did you limit the kinds or amounts of food you eat because of problems with your teeth or denture ?	47 (52.2%)	17 (18.9%)	14 (15.6%)	11 (12.2%)	1 (1.1%)
How often did you have trouble biting or chewing any kinds of food, such as a firm meat or apples ?	32 (35.6%)	22 (22.2%)	19 (21.1%)	9 (10%)	9 (10%)
How often were you able to swallow comfortably ?	30 (33.3%)	36 (40%)	10 (11.1%)	6 (6.7%)	6 (6.7%)
How often have your teeth or dentures prevented you from speaking the way you wanted ?	60 (66.7%)	8 (8.9%)	10 (11.1%)	5 (5.6%)	5 (5.6%)
How often were you able to eat anything without feeling discomfort ?	18 (20%)	25 (27.8%)	17 (18.9%)	17 (18.9%)	13 (14.4%)
How often did you limit contacts with people because of the condition of your teeth or dentures ?	68 (75.6%)	5 (5.6%)	10 (11.1%)	3 (3.3%)	4 (4.4%)
How often were you pleased or happy with the appearance of your teeth, gums or dentures ?	11 (12.2%)	36 (40%)	19 (21.1%)	15 (16.7%)	9 (10%)
How often did you use medication to relieve pain or discomfort around your mouth ?	31 (34.4%)	20 (22.2%)	25 (27.8%)	8 (8.9%)	6 (6.7%)
How often were you worried or concerned about the problems with your teeth, gums or dentures ?	27 (30%)	15 (16.7%)	24 (26.7%)	18 (20%)	6 (6.7%)
How often did you feel nervous or self-conscious because of problems with your teeth, gums or dentures ?	35 (38.9%)	18 (20%)	24 (26.7%)	10 (11.1%)	3 (3.3%)
How often did you feel uncomfortable eating in front of people because of problems with your teeth or dentures?	66 (73.3%)	7 (7.8%)	11 (12.2%)	1 (1.1%)	5 (5.6%)
How often were your teeth or gums sensitive to hot, cold or sweet foods ?	22 (24.4%)	18 (20%)	30 (33.3%)	11 (12.2%)	9 (10%)

Add-GOHAI score: mean = 45.53 (8.41), minimum = 17, maximum = 60

Approximately 29% of the patients declared that they limited the amount of food eaten (sometimes more) and 41% had problems chewing meat or apples due to problems with their teeth, prostheses or gums. One patient in four could swallow comfortably while half said that they could eat everything without discomfort. The majority of the patients (77.7%) said that their teeth or prostheses did not prevent them from speaking. Among the patients, 47.8% were not satisfied with the appearance of their teeth but only 18.8% limited contact with people for this reason. Roughly one fifth (18.9%) felt embarrassed eating in front of others and 53.4% worried or felt uncomfortable because of problems with their mouth. Almost 45% took medication for pain relief, and sensitivity to cold or heat caused discomfort for 55.5% of participants. The mean Additive GOHAI score was 45.53 (SD = 8.41) with a minimum of 17 and a maximum of 60. Approximately half of the sample had a GOHAI score between 41 and 50.

### Reliability

Cronbach’s alpha (α = 0.82) showed a high degree of internal consistency and homogeneity between items (Table 2). Item-scale correlations varied between 0.22 and 0.82. The highest coefficients were obtained for items (*X*2, X10, X11) and the lowest coefficient was obtained for items (X3, X5, X7). When items with low item-scale correlation values (X3, X5, X7) were removed, the value of Cronbach’s α increased to 0.87.

**Table 2:** Item-scale correlations for the GOHAI items

GOHAI items	1	2	3	4	5	6	7	8	9	10	11	12
Item-scale correlations	0.579	0.745	0.380	0.635	0.219	0.688	0.389	0.597	0.699	0.819	0.740	0.516

Cronbach’s α = 0.82

The reproducibility of the GOHAI index was verified with a Pearson correlation coefficient of 0.65 and no significant difference between the two mean GOHAI scores (*p*-value = 0.09) with the Wilcoxon paired test.

### Validity

#### Predictive validity

The GOHAI score was higher in younger patients (*p* = 0.03), in patients who declared they brushed their teeth daily (*p* = 0.02) and with satisfaction with oral health (*p* < 0.01). The GOHAI score did not vary depending on the education level, the place of residence or the participant’s sex. Neither dental attendance, nor duration of the mental illness nor smoking habits influenced the GOHAI score (Table 3).

**Table 3:** Predictive validity

Variable	Mean Add-GOHAI [CI (95%)]	Significance
Satisfaction with oral health		
Yes (*n* = 35)	50.93 [50.87; 50.99]	<0.01
No (*n* = 55)	42.10 [42.06; 42.14]	
Duration of illness		
≤ 18 years (*n* = 43)	47.12 [47.07; 47.17]	NS
18 ans et plus (*n* = 47)	44.08 [44.02; 44.15]	
How long since last dental visit?		
≤ 6 months (*n* = 35)	46.55 [46.46; 46.64]	NS
6 months or more (*n* = 54)	44.89 [44.85; 44.93]	
Smoking habit		
Yes (*n* = 48)	44.38 [44.31; 44.45]	NS
No (*n* = 42)	46.54 [46.49; 46.59]	
Frequency of tooth brushing		
Never to Occasionally (*n* = 22)	42.76 [42.59; 42.95]	0.02
Always (*n* = 58)	47.18 [47.15; 47.22]	
Age		
≤ 40 year (*n* = 27)	48.68 [48.59; 48.77]	0.03
> 40 year (*n* = 63)	44.19 [44.15; 44.22]	
Sex		
Male (*n* = 58)	45.97 [45.93; 46.00]	NS
Female (*n* = 32)	44.74 [44.63; 44.86]	
High school education		
Primary school or less (*n* = 19)	42.43 [42.15; 42.71]	NS
Secondary or higher (*n* = 70)	46.51 [46.49; 46.54]	
Residence		
With the Family (*n* = 21)	47.05 [46.79;47.30]	NS
Autonomous (*n* = 61)	45.15 [45.11; 45.18]	
Hospitalized (*n* = 8)	44.49 [43.99; 44.98]	

*NS* Not significant (*p* > 0.05), *CI* Confidence Interval

#### Concurrent validity

A lower GOHAI score was associated with higher DMFT and OHI-S values (*p* = 0.0002). The GOHAI score was related to the number of missing teeth and to the number of filled teeth (*p* < 0.05) (Table 4).

**Table 4:** Concurrent validity

Variable	Mean Add-GOHAI [CI (95%)]	Significance	Mean ± SD
Number of carious teeth (DT)			1.98 ± 2.59
DT < 2 (*n* = 50)	46.45 [45.40;46.50]	NS	
DT ≥ 2 (*n* = 40)	44.39 [44.33;44.46]		
Number of missing teeth (MT)			7.89 ± 9.15
MT < 4 (*n* = 41)	50.17 [50.12; 50.21]	*p* < 0.01	
MT ≥ 4 (*n* = 49)	41.66 [41.61; 41.71]		
Number of filled teeth (FT)			6.72 ± 6.72
FT ≤ 5 (*n* = 41)	43.24 [43.17;43.32]	*p* = 0.02	
FT > 5 (*n* = 49)	47.45 [47.41;47.49]		
DMFT			16.59 ± 8.11
DMFT ≤13 (*n* = 33)	49.36 [49.29;49.43]	*p* = 0.0002	
13 < DMFT ≤ 20 (*n* = 25)	46.81 [46.69; 46.92]		
DMFT >20 (*n* = 32)	40.59 [40.48; 40.71]		
OHI-S			2.37 ± 1.34
OHI-S ≤1.5 (*n* = 15)	49.53 [49.45; 49.62]	*p* = 0.02	
1.5 < OHI-S ≤ 3 (*n* = 28)	46.25 [45.13; 46.36]		
OHI-S > 3 (*n* = 23)	42.16 [41.96; 42.36]		

*NS* Not significant (*p* > 0.05), *CI* Confidence Interval, *SD* Standard Deviation

#### Known-groups validity

They were no significant differences between the mean GOHAI score for in-patients 45.16 [CI(95%): 40.14; 49.37] and that for out-patients 45.78 [CI(95%): 43.79; 47.70], (*p* > 0.0.5).

#### Construct validity

**Fig. 1:**
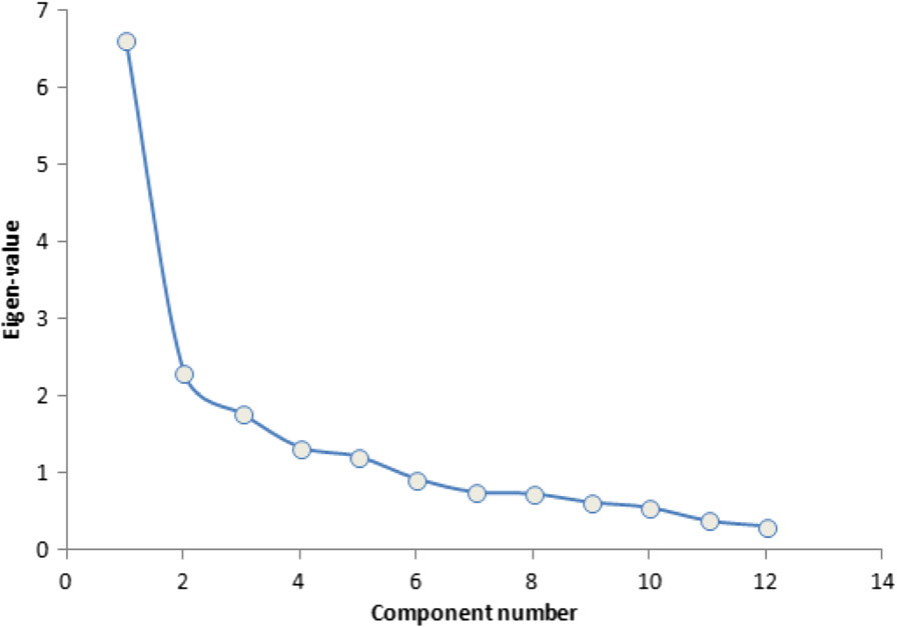
Scree Plot-GOHAI

The results of the factor analysis for GOHAI are given in Table 5. Exploratory factor analysis revealed three-factor structure on the basis of eingenvalue > 1. On factor explained 37.69% of the total variance. Two 50.82% and three-factor accounted for the 60.94% of the variance (scree plot; Fig. 1). The KMO measure of simple adequacy was 0.81 (KMO > 0.5) and the Bartlett’s sphericity test was 378.64 with 66° of freedom (*p* < 0.000).

**Table 5:** Component matrix of the factorial analysis of the GOHAI in the sample of schizophrenic persons

	Component		
	1	2	3
1	0.465	0.233	0.322
2	0.532	0.301	0.152
3	0.179	0.249	0.436
4	0.441	0.249	0.114
5	0.054	0.582	0.193
6	0.598	0.519	0.055
7	0.231	0.457	0.190
8	0.660	0.133	0.173
9	0.689	0.459	0.518
10	0.756	0.538	0.296
11	0.673	0.209	0.101
12	0.407	0.055	0.385

## Discussion

The objective of this study was to evaluate the psychometric characteristics of the GOHAI scale in a French representative sample (Côte d’Or) of patients with schizophrenia.

The GOHAI is widely used in clinical or epidemiological studies worldwide, as it is available for use in different languages [41]. Nevertheless, the reliability or validity of the existing versions might vary due to cultural, medical or linguistic characteristics of the study population [42].

In our study, the reliability was confirmed (α = 0.82). Cronbach’s α was slightly lower than that found in a French general population (α = 0.87) but was within the range (0.74-0.87) observed in other validation studies [8, 9, 41–44]. When three questions (X3, X5, X7) were removed, Cronbach’s α increased to 0.87. A rewording of these questions should be considered as in the Chinese version of the GOHAI, where question X3 was reformulated negatively to improve understanding [43]. In schizophrenic patients, the use of questions worded positively and negatively (X3, X5) and the concept evoked in question X7 may have been particularly misunderstood.

Most participants were unable to clearly distinguish between ‘Always’ and ‘Very often’,‘Seldom’ and ‘Never’. A simple 3-point likert scale was used as cited by Atchison [45], where the English equivalents of ‘Always’, ‘Very Often’ and ‘Often’ were clubbed together as ‘Always’; ‘Seldom’ and ‘Never’ were clubbed into ‘Never’, thereby giving ‘Always’, ‘Sometimes’ and ‘Never’ as the three options. The resulting mean GOHAI score of their study, according to the new scoring system, was higher than the current study [45, 46]. The 3-point system should be explored with schizophrenic persons for better understanding.

The predictive and concurrent validity of the GOHAI were confirmed. The GOHAI score was significantly higher for younger patients. In some other studies, no significant relationship was found between age and the GOHAI score [9, 44]. This can be explained either by age groups that were too narrow [47] or by an adaptation to poor oral health among older people [48]. Another potential reason for this difference may be that the relationship between oral health and age may be different in persons with schizophrenia than in the general population.

For the known-groups validity, there were no significant differences between the mean GOHAI score for out- or in-patients in our sample. The perception of OHrQOL was found to be dependent on the particular psychiatric diagnosis (bi-polar disease, mood disorders or schizophrenia) [49]. The extent of dental disease was directly associated with the severity of the positive and negative symptoms in patients with severe mental illness (SMI) [50–54]. However, schizophrenia is a complex disease which presents distinctive clinical features that distinguish it from bipolar disease or mood disorders. The treatment can vary in intensity and diversity depending on the stage of the disease, its duration and its severity, and on the patients’ social characteristics and environment [55, 56].

In our representative sample of schizophrenic persons, the difference in perception of OHrQOl did not differ between out- or in-patients.

Therefore, this known-groups validity needs to be confirmed or invalidated in future studies.

While only one factor emerged in factor analysis in the original English GOHAI version [8], three factors emerged with schizophenic persons when using the principal components with eigenvalues greater than one. In Swedish [57] and Chinese [43] version the factor analysis also revealed three factors. The reason for this may be that the complexity of the OHrQol is refleted in the separate items of GOHAI. It possible means that the participants do not differentiate among psychological, functional, or behavioral impacts and that the perception of the oral health is a global concept.

Our results showed no significant relationship between the duration of mental illness, smoking habits, dental attendance and the GOHAI score. However, the duration of hospitalization (a sign of severe symptoms) or smoking seemed to be associated with poor dental status in patients with schizophrenia [15, 16]. Moreover, the presence of severe symptoms is associated with poor dental health, indicating a late use of dental care [41]. It would thus be necessary to explore more thoroughly these relationships in further research using GOHAI scores or other measures of quality of life in schizophrenic patients.

The literature contains examples of high [8, 43] and low correlations [13] between GOHAI scores and clinical observations, except for the number of decayed teeth, which is closely related to OHrQOL scores. Furthermore, the level of education or the place of residence often correlates with GOHAI scores [8, 43, 58], but for schizophrenic patients, we found no significant differences. In our study, several clinical measurements were related to the GOHAI score, indicating that the perception of the patients about their oral health was clearly associated with their objective dental status.

In this study, schizophrenic patients seemed to be less satisfied with the condition of their teeth and to experience a higher impact of their dental health than did the French general population [9]. These differences can be explained by a worse dental status (namely a higher number of untreated carious lesions) [30] and a poor oral hygiene as compared with the general population. 50% of patients with schizophrenia never brush their teeth (36% in our sample) while 60% of the control groups brush their teeth once or twice daily [59]. Another study showed less frequent tooth brushing was associated with a greater DMFT, less frequent tooth brushing was negatively related with dental condition in patients with schizophrenia [60].

Conversely, compared to the literature, schizophrenic patients described fewer symptoms, such as sensitivity, compared with the general population [9]. The confusion brought on by mental illness means, for those who are most perturbed by the disease in particular, that the patient does not have a good perception of his or her needs or of pain and can thus postpone dental consultations and seeking treatment [6]. Dry mouth was the chief complaint among 40% of the psychiatric patients while dental pain was the main complaint among 60% of the control group [59]. The negligence in self-care of schizophrenic patient seems to be more influenced by symptoms such as lack of concern with personal health, lack of motivation and apathy [61]. However, in the schizophrenic population, the GOHAI scale is able to express the patients’ perception and subjective evaluation of OHrQOl.

Even though the majority of patients with schizophrenia do not have severe or persistent symptoms all the time or severe side effects of medication, further studies are needed to better understand their perception of OHrQOL. To this end, the relationship with clinical manifestations of the side effects of antipsychotics, like drooling, trismus, facial muscle pain, myasthenia or dyskinesia (tremors), must be investigated in future studies [62, 63]. Moreover, the impact of the deterioration or rehabilitation of the dental status on self-esteem or on stigma and on social functioning could be evaluated according to modifications of perceived oral health.

A few limitations of the present study must be noticed. First, only 34% of the patients contacted were included. This is a potential selection bias. It was difficult to include schizophrenic persons in the study. Some potential subjects were unable to cooperate due to their psychiatric illness, some were lost to follow-up, others were excluded for diagnostic error (not schizophrenia), and some had died. Furthermore, we can suppose that patients who do not agree to participate in these studies are likely to be unwilling to have a dental check-up because of their poor oral health.

Second, the test-retest reliability was performed with a consecutive rather than random sample.

Third, all of the investigations were conducted by only one investigator.

Fourth, our results are representative of the Côte d’Or department, and the relatively small number of participants limits the robustness of the results. These findings need to be replicated in larger groups of schizophrenic persons.

Therefore, a potential variety bias in our sample cannot be completely excluded.

## Conclusions

This is the first study to establish the reliability and validity of the GOHAI scale in a French representative sample of patients with schizophrenia, thus making it a suitable tool to measure OHrQOL and its multiple aspects in this population. Having a validated scale to evaluate OHrQOl in patients with schizophrenia provides a positive step towards future multidimensional research in mental and physical well-being to this group. The evaluation of self-perceived oral health is necessary to help caregivers and researchers to develop ways to improve oral and overall health in schizophrenic patients. In the future the GOHAI could support research in assessment schizophrenia specific oral health scale.

## Abbreviations

DMFT: Decayed, Missing, or Filled teeth
EFA: Exploratory factorial analysis
GOHAI: General oral health assessment index
ICC: Intra class correlation coefficient
OHI-S: Simplified oral hygiene index
OHrQOL: Oral health related quality of life
QLS: Quality-of-life scale
S QOL: Schizophrenia quality of life scale
SES: Socio-economic status
SES: Socio-economic status
SMI: Severe mental illness
